# Testing the Strength of Hospital Accreditation as a Signal of the Quality of Care in Romania: Do Patients’ and Health Professionals’ Perceptions Align?

**DOI:** 10.3390/healthcare8030349

**Published:** 2020-09-19

**Authors:** Elena Druică, Bingyi Wu, Vasile Cepoi, Viorel Mihăilă, Marin Burcea

**Affiliations:** 1 Centre for Applied Behavioral Economics, Faculty of Business and Administration, University of Bucharest, 030018 Bucharest, Romania; viorel.mihaila@faa.unibuc.ro; 2 College of Business, University of Texas at San Antonio, NPB 2.146, San Antonio, TX 78249, USA; bingyi.wu@utsa.edu; 3 The Romanian Authority for Quality Assurance in Healthcare, 060022 Bucharest, Romania; vasile.cepoi@anmcs.gov.ro; 4 Faculty of Business and Administration, University of Bucharest, 030018 Bucharest, Romania; marin.burcea@faa.unibuc.ro

**Keywords:** hospital accreditation, patient freedom of choice law, quality of healthcare

## Abstract

Hospital accreditation, as a quality signal, is gaining its popularity among low- and middle-income countries, such as Romania, despite its costly nature. Nevertheless, its effectiveness as a quality signal in driving patients’ choice of hospital services remains unclear. In this study, we intend to empirically explore the perceptions of both healthcare professionals and patients toward Romanian hospital accreditation and identify perception gaps between the two parties. Exploratory and confirmatory factor analyses were carried out to extract the latent constructs of health professionals’ perceived effects of hospital accreditation. The Wilcoxon rank-sum test and Kruskal–Wallis test were used to identify correlations between patients’ sociodemographic characteristics and their behavioral intentions when confronted with low-quality services. We found that health professionals believe that hospital accreditation plays a positive role in improving patient satisfaction, institutional reputation, and healthcare services quality. However, we found a lack of awareness of hospital accreditation status among patients, indicating the existence of the perception gap of the accreditation effectiveness as a market signal. Our results suggest that the effect of interpersonal trust in current service providers may distract patients from the accreditation status. Our study provides important practical implications for Romanian hospitals on enhancing the quality of accreditation signal and suggests practical interventions.

## 1. Introduction

The need for reforming the Romanian healthcare system and improving the quality of care has been an important concern for the last 30 years, since the fall of the communist regime [1,2]. Service quality improvement through hospital accreditation is one of the prerequisites for achieving and maintaining good performance of the health system [3]. Initially developed in the USA [4], the available programs increased around the world with a particular development in Europe [5] but also beyond it [6]. Recently, accreditation has gained popularity in enhancing healthcare quality in low- and middle-income countries [7]. International organizations such as the World Health Organization (through the National Quality Policy and Strategy, or the WHO Patient Safety Program), the EU Health Systems Performance Assessment Group, the European Observatory on Health Systems and Policies, or The International Society for Quality in Health Care (ISQua) have already provided nations reliable standards for health quality accreditations. However, despite developing in a more general practice at the EU level, the accreditation remains to an important extent a national-led decision based on an imported model.

Romania went through the first wave of hospital accreditation in 2018. However, despite the importance of the outcomes and their relevance for practical interventions, the studies on the matter remain scarce, covering ethical dimensions [8,9], patients’ rights and communication [10], or governance-related determinants of hospital performance [11]. There is no research assessing the effects of hospital accreditation on both health professionals and patients.

Our study aims to fill this space and empirically analyze the perception gap between patients and health professionals on the effects of hospital accreditation, using data collected from two different studies. The first study surveys 5294 health practitioners, medical doctors, and nurses working in 340 hospitals from 190 Romanian localities, while the second study investigates a country—a representative sample of 1500 Romanian patients. We assess the health professionals’ perception regarding the effectiveness of hospital accreditation in directing a patient’s choice for hospital services. We measure patients’ awareness regarding hospital accreditation, then analyze preferred courses of action that patients may take when confronted with low-quality hospitals. Last, we explore the health professionals’ perspective regarding the effects of hospital accreditation in terms of quality improvement, patients’ satisfaction, and hospital reputation. 

The contribution of the paper is multifold. First, there is no other study in Romania assessing both the perceived effects of hospital accreditation among health professionals and the self-reported knowledge on the matter among patients. Second, we explore the perceived strength of accreditation as a quality signal in Romania in the view of both health professionals and patients and compare the results. Last but not least, we advance practical recommendations.

The rest of the paper is organized as follows: the next section summarizes the existing literature by discussing the main approaches of hospital accreditation. Section 3 presents the data and briefly summarizes the method. Section 4 presents the results, while Section 5 discusses the findings and the implications of this research. We conclude with Section 6 and present the limits of the research.

## 2. Background

Hospital accreditation programs are defined as systematic assessments of hospitals against a series of quality standards [12] and have been pursued as an intervention for hospitals to improve their quality of healthcare outcomes (e.g., the process of care and patient satisfaction). Although it is evidently favored in terms of the positive results, the accreditation processes can be costly. First, there are implementation costs for obtaining accreditations associated with infrastructure, research and development, maintenance, and permanent professional training and development, varying from one hospital to another. Second, there are maintenance costs. According to the Romanian Authority for Quality Management in Healthcare [13], these costs might vary from 145 to 745 euros/month for the accreditation program, with a monthly average of 260 euros. For instance, for a hospital with 1 unit, at the local level, the fee might be 41,880 RON (8637 EUR); for a hospital with seven units, local level, 80,886 RON (16,680 EUR); and for a university hospital with 22 units, regional level, the fee could be as much as 194,349 RON (40,079 EUR). Hospitals shall pay the total amount in three installments over the entire evaluation timeline—the first 30%, the second 40%, and the remaining 30%.

Although these costs can be essential barriers against accreditation, the noncompliance costs, reaching up to 20% of the hospital resources, have adverse effects on hospital reputation and increase costs originating in, for example, medical errors, out–of–date drugs and materials, and temporary out-of-service medical technologies. The rationale behind the adoption of accreditation assumes that quality assurance costs are lower than potential costs of noncompliance or that benefits of accreditation are significant. Nevertheless, there is only sparse evidence on the effectiveness of such a measure.

At the EU level, the impact of the existing perspectives at the national level is relatively marginal, taking into account that “Community action in the field of public health shall fully respect the responsibilities of the Member States for the organization and delivery of health services and medical care”, as stated in Article 152 of the Treaty [14,15]. A comprehensive analysis of the healthcare quality in Europe [16] signals that the main denominator for accreditation through Europe is external assessment. Although there is not yet a general agreement on the proven efficacy of the accreditation, there is a widespread belief that this process will improve the quality of care. For policy-makers, in constant need of reassurance that quality in healthcare is under control, the accreditation is a catchy word. However, at the EU level, “an overarching approach that would coordinate the various dimensions of healthcare quality is missing” [16] but, in the meantime, quality in healthcare seems to become an international political priority supported by a web of international organizations.

Several studies suggested that accreditation has a positive impact on the quality of healthcare deliverables and should be supported at the managerial level. For example, Thornlow and Merwin [17] found that preventive protocols included in accreditation measurements reduced certain adverse events (e.g., infections and decubiti). Also, in their systematic review of the impact of accreditation, Alkhenizen and Shaw [18] showed that most earlier studies provided evidence that both general and subspecialty accreditation programs significantly improve the process of care and clinical outcomes. Moreover, a prior study conducted in Jordan to compare the impacts of accredited and nonaccredited hospitals on patient satisfaction provided supports that accreditation significantly enhances patient satisfaction [19]. Moreover, a recent review on the impact of hospital accreditation suggests that accreditation may have a positive impact on healthcare quality dimensions such as efficiency, safety, timeliness, and patient-centeredness. However, such a positive impact needs to be interpreted with caution due to the shortcomings in methodologies used [20].

In contrast, some other studies were unable to identify the significant association between accreditation and quality of healthcare services [12,21]. More surprisingly, a negative relationship between accreditation scores and patients’ satisfaction levels was found in recent studies of US and Iran hospital accreditation systems. Their findings suggest that the increased requirements of accreditation programs in hospitals’ managerial processes may burden employees’ workloads and sacrifice the quality of medical services, indicating that the incompatibility between accreditation standards and patients’ needs might contribute to the negative association [18,19]. A recent study [20] points out the fact that accreditation might have a positive impact on efficiency, safety, and effectiveness, mixed results on patient-centeredness, and moderate impact on timeliness dimensions but has no effect on access indicators. Moreover, for accreditation implementation to be effective in improving patient satisfaction, ongoing evaluations on service quality are recommended to be carried out [22]. In addition to the possible impact on the quality of healthcare services, does getting accredited make hospitals more profitable? How does accreditation influence patients’ choices of hospitals? A prior study of [23] argued that although hospital accreditation acts as a market-signaling device, it might be inefficient as a tool for influencing stakeholders’ behavior for two reasons. Firstly, there is a lack of evidence on the effectiveness of hospital accreditation on quality improvement in healthcare services. Secondly, the normalization of accreditation among hospitals occurs when the implementation and maintenance costs of accreditation are so affordable that low-quality hospitals can easily “mimic” the signals of high-quality hospitals. Therefore, the perceived value of accreditations is diluted. The more accredited hospitals, the less effective the accreditation to the market will be perceived. Therefore, accreditation ceases to act as a differentiating position in the hospitals’ market. In this case, the strength of the accreditation signal may determine the effect of accreditation on influencing patients’ decision-making processes during the selection of hospitals. Moreover, other researchers suggested that there was no evidence indicating the differential impacts of government and independent accreditations on patients’ choices of hospitals in the US [24].

From a managerial perspective, similar to other private or public companies, pursuing hospital accreditations is one of the strategic management approaches for hospitals to polish operational processes, to build institutional reputations, and to send quality signals to the market. Nevertheless, the effectiveness of an accreditation signal is dependent on various motives and barriers. According to the signaling theory [25], the effectiveness of a signaling approach relies on the signaler’s attributes, the signal’s quality, and the receiver’s perception [26]. In other words, for a hospital accreditation signal to be effective, the accredited hospital needs to, first, be honest and deliver expected quality services [27]. Second, the hospital accreditation signal should be observable [28] and fit the patients’ needs [29,30] Third, the hospital accreditation signal can be interpreted appropriately, and the importance of the signal is weighed more than other barriers perceived by receivers [31].

The primary barrier that is likely to prevent patients from choosing or switching to an accredited hospital is the perceived switching costs, which can be specified as perceived procedural costs, perceived relational costs, and perceived financial costs [32]. A prior study shows that as perceived procedural and relational costs of changing hospitals increase, the quality of curing service and interpersonal service become more critical to patient loyalty. In contrast, the perceived financial costs do not have a significant moderating impact on the relationship between the quality of healthcare services and patient loyalty [32]. In a similar vein, a recent study of women’s choice of hospital for childbirth in Florida also found that switching costs account for approximately 40% of the reasons for women to return to the same hospital [33]. Moreover, a prior study suggested that image congruence positively moderates the patients’ trust transfer, indicating that the barrier to switching to other hospitals may also depend on the perceived fitness between the image of hospital specialty and patients’ needs [34].

## 3. Materials and Methods

### 3.1. Data and Software

We used data from two different surveys conducted in 2018 after the first round of hospital accreditation had been completed. The first survey addressed healthcare professionals, while the second survey captured patients’ perspectives on hospital accreditation.

The healthcare professionals’ sample consisted of 5294 respondents reached via an online questionnaire, comprising health professionals working in 340 hospitals located in 190 Romanian localities. The questionnaire was applied online through ANMCS ‘CAPESARO software used to communicate with accredited hospitals. The hospital unit sampling was stratified by type of property (private/public), guardianship authorities, and classification rank. The patients’ sample comprised 1500 respondents, representative of the Romanian population. The sampling was probabilistic, random, and stratified (regional, county level, and village/city level). This sample size provides a margin of error of ± 3% with 95% accuracy. We used a paper and pen approach, and the Romanian Center for Urban and Regional Sociology collected the data. Supplementary Material Tables S1 and S2 show the sociodemographic characteristics of each sample. To analyze our data, we used the R software version 3.6.1. and its interface R Studio.

### 3.2. Questionnaire and Measurement

The questionnaires were applied in the Romanian language, the participation in the study was voluntary, and the research complied with the APA standards and recommendations. They were developed in line with the International Society for Quality in Health Care (ISQua) principles stated in the Guidance on Designing Healthcare External Evaluation Programmes, including Accreditation, and also the Romanian Authority for Quality Assurance in Healthcare (RAQAH) standards. The health professionals’ questionnaire pursued a multidimensional construct, based on content analysis, expert judgment, and review of research in the perception of the hospitals’ accreditation field. The questionnaire was pretested and checked for internal consistency, content validity (panel of experts), and construct validity. We measured the level of agreement using a Likert measurement 1–5. Supplementary Material Tables S3 and S4 present the questions.

### 3.3. Methods

Using both parametric and nonparametric statistical methods for continuous variables to analyze items measured on a 1–5 scale is common practice, rooted in rich literature showing that a 1–5 measurement scale can be transformed into numerical scores [35,36].

First, we conducted an exploratory factor analysis (EFA) to identify potential latent constructs measuring health professionals’ perception regarding the effects of hospital accreditation. Originated in the early 1900s concerning Charles Spearman’s interest in developing the Two-Factor Theory [37], factor analysis is a multivariate statistical technique commonly used in psychological research [38], applied psychology [39], and, more recently, in health-related professions [40]. The functions involved in conducting EFA in this paper are available in the “psych” package in R. Given the non–normal distribution of the items, we used the principal axis as an extraction method and the “onlimin” rotation, although the extraction method “minres” led to similar results [41]. Exploratory factor analysis is a useful strategy for model specification prior to cross-validation with confirmatory factor analysis [42]. Therefore, after extracting the factors, we conducted a confirmatory factor analysis to assess the model’s performance [43]. Confirmatory factor analysis functions are available in the “lavaan” package in R.

To test whether health professionals’ perceive the effects of hospital accreditation as positive, we analyzed the latent constructs identified during the factor analysis stage. First, we used the D’Agostino skewness test [44], available in the “moments” package in R, to check whether the distributions were left- or right-skewed [45]. This test, widely used in research reporting [46], sets as the null hypothesis that the skewness of a specific distribution is 0, while the alternative hypothesis states that it is not. In our case, the D’Agostino test revealed that all three distributions were left-skewed. As such, to test whether the actual location of these distributions was positive, we relied on the nonparametric Wilcoxon rank-sum test [47,48]. In addition, we used the Wilcoxon rank-sum test and the Kruskal–Wallis test [49] to determine the sociodemographic variables that relate to patients’ knowledge and behavioral intentions revealed by our measurements. Both the usual t-test and the ANOVA are robust to normality violations.

## 4. Results

We present the results of two different studies. First, we report how health professionals perceive the effects of accreditation, and to what extent they consider that accreditation signal’s quality in the eyes of family physicians and patients. Second, we explore to what extent patients perceive accreditation as essential in their decision to pursue one hospital or another.

### 4.1. Assessing Health Professionals’ Perception Regarding the Effects of Hospital Accreditation

Table 1 presents the results of an exploratory factor analysis involving the items that measure the perceived effects of accreditation. The value of Cronbach’s Alpha for the health professionals dataset was 0.94, and the Kaiser–Meyer–Olkin statistic was also 0.94. The items loaded in three factors that explained 68.6% cumulative variance. We call these factors “perceived differences in quality” (PDQ henceforth), “actions taken after accreditation” (ATAA henceforth), and “actions to improve quality” (AIQ henceforth).

Our confirmatory factor analysis shows the excellent performance of the model with three factors (CFI 0.979, TLI 0.972, RMSEA 0.068, and SRMR 0.023). Next, we tested whether the distributions of the identified latent constructs tended to be skewed towards positive values.

Table 2 presents the results of two statistical tests, showing that health professionals’ perception regarding the effects of accreditation was positive. The D’Agostino skewness test shows that each of the three distributions is left-skewed; therefore, the three latent constructs are not normally distributed.

Given the vital role played by accreditation in assuring patient safety, we asked the respondents to what extent they believe that family physicians will use this signal to direct patients towards hospitals able to provide services of better quality. A Wilcoxon rank-sum test with continuity correction shows that the actual location of the distribution of answers was lower than 3 (V = 3,005,036, *p*-value < 0.001), a result that proves that health professionals are not confident that hospital accreditation will direct actions from family physicians.

### 4.2. Do Patients Adopt Accreditation as a Quality Signal?

In the following section, we report two perspectives: (1) the health professionals’ perspective on patients’ awareness of the importance of hospital accreditation, and (2) the patients’ self-reported perspective on the importance of accreditation in their decision-making processes during hospital selection.

We asked the respondents in the health professionals’ sample two questions: (1) Do you think that the patients who come for medical services knew whether the hospital was accredited or not?” and (2) “To what extent do you believe that the results of the accreditation process will lead patients to (a) choose better-ranked hospitals, (b) stay with the hospital that they choose, or (c) ask for alternatives?” In the meanwhile, we asked patients (1) whether they were aware that hospitals pursue accreditation, and (2) to what extent they would pursue one of the following if they found that the current hospital did not comply with the accreditation standards: (a) Ask the family physician or the specialist for another option, (b) look for another option themselves, or (c) stay with the option because they trust the health professionals of that hospital.

We found that 63% of respondents in the health professionals’ sample considered that patients did not know whether the hospital aligned with quality standards or not, while 23.4% considered that the patients did know. The results are similar to what patients themselves reported: out of the 1500 respondents in the patients’ sample, only 25.4% were aware that hospitals had undergone an accreditation process. The result indicates a lack of signal visibility.

A chi-square test reveals that patients’ knowledge is related to gender such that men are more informed than women. Patients’ education also plays a significant and positive role, those with more years of schooling being better informed than those with less education. Moreover, patients’ knowledge is related to residence, those in an urban area being more aware of accreditation than those in rural areas (see Table 3).

**Figure 1:**
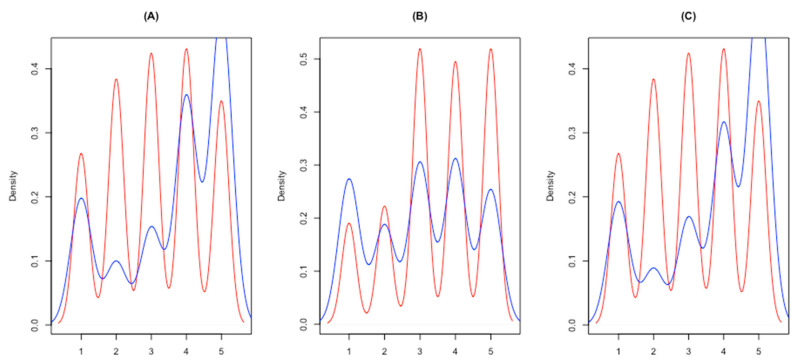
How much do patients value accreditation as a service quality signal? Patients’ perspective (blue) versus what health professionals believe (red) on three matters: (**A**) choose better-ranked hospitals; (**B**) keep the existing option; (**C**) ask the physician for another option.

Regarding the second question, Figure 1 shows a comparison between patients’ answers and health professionals’ beliefs regarding patients’ answers. Health professionals believe that patients are less willing to act in line with the information regarding the quality of the hospital. The first graph (a) shows higher frequencies of lower levels of agreement regarding patients’ initiative of choosing better-ranked hospitals among health professionals.

Also, health professionals’ expectations that patients tend to keep going to a hospital despite its lower accreditation scores are higher than patients’ reported intentions (b). This result can be explained by the perceived level of switching costs. The third graph (c) shows that health professionals expect patients to ask their physicians for better hospital options, to a lesser extent than patients themselves report.

While health professionals’ underestimation of patients’ behavioral intentions when confronted with low-ranked hospitals are apparent, patients’ reported intention deserves particular attention. Figure 1A,C shows that although patients tend to agree that they would look or ask for better options, there is an important share of the respondents who disagree. Besides, Figure 1B shows that keeping the existing option has a nearly uniform distribution that suggests no exact position against low-quality hospitals, as long as they trust in their health professionals. Once again, a possible explanation can be related to switching costs that act as barriers.

An important factor that can explain the differences between these distributions is health services availability. Rural areas and small towns provide less health access and fewer options than larger cities. Table 4 shows, however, that there are no significant differences across residence categories concerning patients’ reported tendency to ask the physician for another option, keep the existing option, or look themselves for a different option. The result holds if the residence is treated as a binary variable (urban–rural) instead of a variable with five categories.

Table 4 also shows no gender differences in patients’ responses to any of the three questions, but education seems to play a role in each case. Higher years of schooling are associated with a higher tendency to ask for alternatives and look for alternatives and a lower tendency to stay with existing suboptimal options. Patients who are aware that hospitals undergo accreditation are more inclined to pursue better options. However, knowing about accreditation does not make a difference in case of the intention of maintaining a possible default option. Age is negatively correlated with patients’ inclination to ask their physicians for another option, negatively correlated with patients’ inclination to look for options themselves, and positively correlated with their inclination towards the status quo.

## 5. Discussion

Our study shows that, overall, health professionals believe that hospital accreditation resulted in positive effects on increased patient satisfaction, better hospital reputation, higher quality of medical care, and improvements in administrative services. These results are in line with previous research that identified the positive effects of hospital accreditation [17,18,19].

We also align with previous research showing that hospital accreditation does not necessarily play a role in patients’ choice of hospital services [23,24]. We found that, despite patients’ self-reported tendency to look for alternatives when facing low-quality healthcare providers, there is also a tendency towards maintaining the option recommended by a physician on the basis that the health professionals working in that hospital are trustworthy.

Our results show that the more educated people are, the less inclined they are to accept low-quality hospital services and the more inclined they are to ask or look for alternatives. Age, on the other hand, has a negative effect on patients’ choice of better-quality services. We suggest that this can be related to the switching costs. Previous literature [8,32,33] shows that older patients stay longer with their current hospitals, and the longer their tenure is, the higher the switching cost is, and in turn, the less likely they will be motivated to switch. Residence is the only factor that does not play a role in patients’ decision-making process during their selection of hospital services. This result is surprising, as it may suggest that the actual access to health services is unrelated to patients’ attitudes and intentions. The result may confirm the previously documented gap between intention and action [50].

In terms of accreditation as a signal, we found that only 25.4% of the patients were aware that hospitals undergo accreditation. The result indicates a lack of signal visibility that may be improved by pursuing information or marketing campaigns. Our results show that an important determinant of accreditation awareness is urban residence. Rural patients are less informed, and the list of hospital service options are limited. Therefore, even if the hospital accreditation signal becomes observable [28], it may not fit a patient’s expectation [29,30] of a better service alternative. Therefore, it would be difficult for these patients to weigh the importance of the signal more than the objective barriers, as previous research suggests [31].

One of the most exciting results shows that health professionals underestimate the strength of the accreditation signal in the eyes of the patients and believe that family physicians will also tend to ignore this information. Although we do not have evidence regarding the exact opinion of the family physicians on this matter, we found that there is no clear tendency among patients against low-quality hospitals as long as they trust in their health professionals. The result points towards several issues that have a long history in Romania [51,52,53]. On the one hand, relying on interpersonal trust instead of trusting formal institutions is a preference that has been developed in Romania in decades of the communist regime [54]. Although a rational adaptive strategy at the individual level, this approach is socially suboptimal as it undermines formal attempts for reforming the healthcare system [55]. No matter how beneficial the hospital accreditation can be in terms of increased quality of hospital services, if the choice is driven by trust in individuals, accreditation may fail in directing patients’ choice towards high-quality hospitals. On the other hand, the mismatch between what patients report and what health professionals believe indicates a structural break in the communication between these categories.

### 5.1. Practical and Managerial Implications

We found that hospital accreditation fails in directing patients’ choice for hospital services, a result that aligns with other findings in the existing literature. However, the perspective presented by our respondents indicates that the first wave of evaluation should not be ignored. Many positive effects were reported in terms of a better quality of services, more efficient and effective resource allocation, clearer strategies for quality improvement, and increased availability of related training and programs. The lessons of this wave of accreditation enable adjustments to current standards to be used in the next evaluation wave. It also indicates the health professionals’ expectations on the accreditation output and substantiates the doctor–patient relationship through the lens of these new shared expectations.

Lastly, our results show that to strengthen hospital accreditation as a signal of service quality, the Romanian officials may design communication campaigns not only to inform patients about what accreditation is but also to build trust that the result of the evaluation reflects the reality of the medical services provided by hospitals.

A workable “trust repository” should be built to take full advantage of the accreditation and benefit the accredited institutions, health professionals, and patients. Accreditations programs should address the representation gap thoroughly within the web of existing meaning for the administrative regulating bodies, hospitals, health professionals, and patients.

### 5.2. Limitations

Our research is not without limitations. First, the health professional sample, although large and covering a significant number of hospitals, is a convenience sample. Until the study is replicated using a representative sample, we cannot entirely rule out the self-selection bias that the health professionals who perceive the positive effects of accreditation are more likely to participate in the research. The second significant limitation lies in the subjective nature of our data. We measured self-reported intended behaviors, or perception, which may impede the understanding of how patients will act in reality. It is not only that previous research shows that behavioral intention and actual behavior correlate less than 30% [56], but our result showing that residence does not have an impact on patients’ behavioral intentions supports this limitation. Future research may address these aspects and further explore the influential determinants of hospital accreditation adoption as a quality signal that drives patient choice.

## 6. Conclusions

Overall, the results of our study show that health professionals believe the positive effects of hospital accreditation on patients’ satisfaction, institutions’ reputation, and the quality of healthcare and administrative services of their hospitals. Our results also found an apparent lack of awareness of hospital accreditation status among patients. In fact, our study indicates that the effect of interpersonal trust in current healthcare service providers may distract patients from the accreditation status as a quality signal. The lack of awareness of hospital accreditation status among patients, indicating the perception gap of the accreditation effectiveness as a market signal between patients and hospital professionals, will hinder the development and improvement of healthcare services, which, in turn, could be detrimental to the citizens’ welfare. The positive effect of hospital accreditation programs on the quality of healthcare services should be rewarded with patients’ attention and supportive market reaction to establish a positive feedback loop, which could further encourage high-quality healthcare practices among low- and middle-income countries (LMIC), such as Romania. Therefore, our study points out the importance for hospitals to include effective communication events that not only educate patients about the evaluation process of accreditation programs but also enhance the fitness and trustworthiness of accreditation as a quality signal in their future plans.

## Figures and Tables

**Table 1:** The results of exploratory factor analysis—the health professionals’ dataset.

Item (Measurement: Likert 1–5)	Perceived Differences in Quality (PDQ)	Actions Taken after Accreditation (ATAA)	Actions to Improve Quality (AIQ)
Increased patients’ satisfaction with medical services	0.753		
Increased quality of administrative services	0.735		
Increased quality of medical services	0.914		
Better hospital reputation	0.748		
Better resource allocation to improve the quality of medical care		0.859	
Developing a clear strategy to improve quality		0.922	
Support to remedy improvable aspects identified during the evaluation		0.833	
Support to improve the hospital’s reputation		0.715	
Training to identify and implement measures to increase the quality			0.806
Ongoing monitoring of patients’ feedback regarding the quality of received medical care			0.840
Other concrete actions to improve the quality of care			0.897

**Table 2:** Statistical tests proving that the health professionals’ perception regarding the effects of hospital accreditation was positive.

Variable	The D’Agostino Skewness Test	Wilcoxon Rank-Sum Test	Conclusion
PDQ	skew = −0.568, z = −15.762, *p* < 0.001	V = 7,313,577 *p* < 0.001	The distribution of PDQ has a negative skewness, and the true location is positive
ATAA	skew = −0.814, z = −21.369, *p* < 0.001	V = 7,713,139 *p* < 0.001	The distribution of ATAA has a negative skewness, and the true location is positive
AIQ	skew = −0.768, z = −20.378, *p* < 0.001	V = 7,593,746 *p* < 0.001	The distribution of AIQ has a negative skewness, and the true location is positive

**Table 3:** Patients’ knowledge about hospital accreditation depends on sociodemographic characteristics.

Chi-Square Test	Are Patients Aware of Hospital Accreditation?	Differences by Predictor
Gender	X-squared = 4.54, *p*-value = 0.033	Yes
Education	X-squared = 53.72, *p*-value < 0.001	Yes
Residence	X-squared = 6.99, *p*-value = 0.008	Yes

**Table 4:** Patients’ self-reported behavioral intentions by sociodemographic characteristics.

Behavioral Intention/Predictors	(a) Ask the Family Physician or the Specialist for Another Option	(b) Look for Another Option Themselves	(c) Stay with the Option Because They Trust the Health Professionals of That Hospital
**Gender**	W = 236960, *p*-value = 0.216	W = 239288, *p*-value = 0.869	W = 222936, *p*-value = 0.646
**Education**	Kruskal–Wallis chi-sq = 6.4695, *p*-value = 0.04 *	Kruskal–Wallis chi-sq = 38.813, *p* < 0.001 ***	Kruskal–Wallis chi-sq = 24.483, *p* < 0.001 ***
**Residence**	Kruskal–Wallis chi-sq = 7.3803, *p*-value = 0.117	Kruskal–Wallis chi-sq = 6.307, *p*-value = 0.177	Kruskal–Wallis chi-sq = 5.613, *p*-value = 0.23
**Aware of hospital accreditation**	Wilcoxon test W = 226992, *p* < 0.001 ***	Wilcoxon test W = 216884 *p* < 0.001 ***	Wilcoxon test W = 184218, *p*-value = 0.975
**Age**	Spearman correlation: −12% *p* < 0.001 ***	Spearman correlation: −20% *p* < 0.001 ***	Spearman correlation: 22.3% *p* < 0.001 ***

* *p* < 0.05; ** *p* < 0.01; *** *p* < 0.001.

## Supplementary Materials

The following are available online at https://www.mdpi.com/2227-9032/8/3/349/s1, Table S1: Descriptive statistics–health professionals’ sample, Table S2: Descriptive statistics–patients’ sample; Table S3: Questions in the health professionals’ sample; Table S4: Questions in the patients’ sample.
