# Supplementary material for: Testing the Strength of Hospital Accreditation as a Signal of the Quality of Care in Romania: Do Patients’ and Health Professionals’ Perceptions Align?
Source: Healthcare (Basel). 2020 Sep 19;8(3):349. doi: 10.3390/healthcare8030349 (PMC7551154; doi:10.3390/healthcare8030349)
Supplement: Supplementary file 1 [file healthcare-08-00349-s001.pdf]

Supplementary material

Table S1. Descriptive statistics – health professionals' sample.

Variable	Frequency	Variable	Frequency
		**Hospital type**	
**Gender**		Chronic diseases	4.8%
Female	83.7%	General	54.5%
Male	16.3%	Specialty	20.7%
		Emergency	19.1%
		**Hospital coverage**	
**Job position**		Local	67.6%
Medical doctor	26.2%	County - level	16.8%
Nurse	73.8%	Regional	11.8%
		Unspecified	1.3%
		**Hospital rank***	
		I	3.4%
		I M	3.8%
**Hospital ownership**		II	5.7%
Public	91.3%	II M	6.2%
Private	7.7%	III	12.1%
		IV	49.9%
		V	14.9%
		Unclassified	3.1%

* Hospital rank according to the "Order no. 10/2018 regarding the approval of the classes of accreditation of the healthcare units with beds corresponding to the cycle II of accreditation, Official Gazette no. 95 from January 31st, 2018": I - accredited (a total score of conformity with the Romanian Authority for Quality Assurance in Healthcare henceforth RAQAH standards of at least 90% after the evaluation); II - accredited with recommendations (at least 70% conformity with the standards of the RAQAH); III - accredited with reserves (at least 51% conformity with the standards of the RAQAH); IV - accredited with low trust (at least 51% conformity with the standards of the RAQAH + less than 30% from the parameters scored by (-10) applicable to the respective healthcare unit); V - is made for a period of mostly 6 months from the reception of the accreditation report, during which a decision to extend the process of accreditation is released and needs the fulfillment by the healthcare units with beds of the new criteria and cumulative conditions.

Table S2. Descriptive statistics – patients' sample.

Variable	Frequency	Variable	Frequency
		**Education**	
**Gender**		Maximum 10 years	30.5%
Female	58.9%	High school	27.5%
Male	41.1%	Vocational school	26.7%
		Bachelor	12.8%
		Master	2.4%
**Civil status**			
Married	61.9%	**Occupation Sector**	
Divorced	5.8%	Public	13.5%
Unmarried	14.0%	Private	28.2%
Consensual union	2.1%	Do not work	58.3%
Other	15.9%		
**Social status**		**Home place (# inhabitants)**	
Similar to other families	61.3%	Village	58.7%
Above average	15.0%	100-200	7.9%
Among the wealthiest	0.8%	30-100	8.9%
Among the poorest	3.5%	>200	16.9%
Under average	16.0%	<30	7.5%

Table S3: Questions in the health professionals' sample

No. crt.	Question	Item	Latent construct
1.	To what extent do you think that the following effects occurred after accreditation?<br><br>Measurement: 1 – 5; 1 = total disagreement; 5 = Total agreement	Increased patients' satisfaction with medical services	"Perceived differences in quality" (PDQ).
		Increased quality of administrative services	
		Increased quality of medical services	
		Better hospital reputation	
2.	To what extent do you agree that after accreditation the following happened?<br><br>Measurement: 1 – 5; 1 = total disagreement; 5 = Total agreement	Better resource allocation to improve the quality of medical care	"Actions taken after accreditation" (ATAA).
		Developing a clear strategy to improve quality	
		Support to remedy improvable aspects identified during evaluation	
		Support to improve hospital's reputation	
3.	To what extent the following type of initiatives have been taken to support the employees after hospital accreditation?<br><br>Measurement: 1 – 5; 1 = total disagreement; 5 = Total agreement	Training to identify and implement measures to increase quality	"Actions to improve quality" (AIQ)
		Ongoing monitoring of patients' feedback regarding the quality of received medical care	
		Other concrete actions to improve quality of care	
4.	Do you think that the patients who come for medical services knew whether the hospital was accredited or not?<br><br>Measurement: Yes/No		Not part of a latent construct
5.	To what extent do you believe that the results of the accreditation process will determine patients to:<br><br>Measurement: 1 – 5; 1 = total disagreement; 5 = Total agreement	(a) choose better ranked hospitals	Not part of a latent construct
		(b) stay with the hospital that they choose;	
		(c) ask for alternatives	
6.	To what extent do you believe that family physicians will use this signal to direct patients towards hospitals able to provide services of a better quality<br><br>Measurement: 1 – 5; 1 = total disagreement; 5 = Total agreement		Not part of a latent construct

Table S4: Questions in the patients' sample

<table>
<tr><th>Question</th><th>Item</th><th>Latent construct</th></tr>
<tr><td colspan="2">Are you aware that the Romanian hospitals pursue accreditation?

Measurement: Yes/No</td><td>Not part of a latent construct</td></tr>
<tr><td>To what extent would you pursue one of the following, if you found that the hospital you are sent to does not comply with the accreditation standards?

Measurement: 1 – 5; 1 = total disagreement; 5 = Total agreement</td><td>(a) ask the family physician or the specialist for another option;
(b) look for another option yourself;
(c) stay with the option because you trust the health professionals of that hospital</td><td>Not part of a latent construct</td></tr>
</table>
